# Supplementary material for: Highly active enzymes by automated combinatorial backbone assembly and sequence design
Source: Nat Commun. 2018 Jul 17;9:2780. doi: 10.1038/s41467-018-05205-5 (PMC6050298; doi:10.1038/s41467-018-05205-5)
Supplement: Supplementary file 1 — Supplementary Information [file 41467_2018_5205_MOESM1_ESM.pdf]

Highly active enzymes by automated combinatorial backbone assembly and sequence design

Lapidoth et al

Supplementary Figures

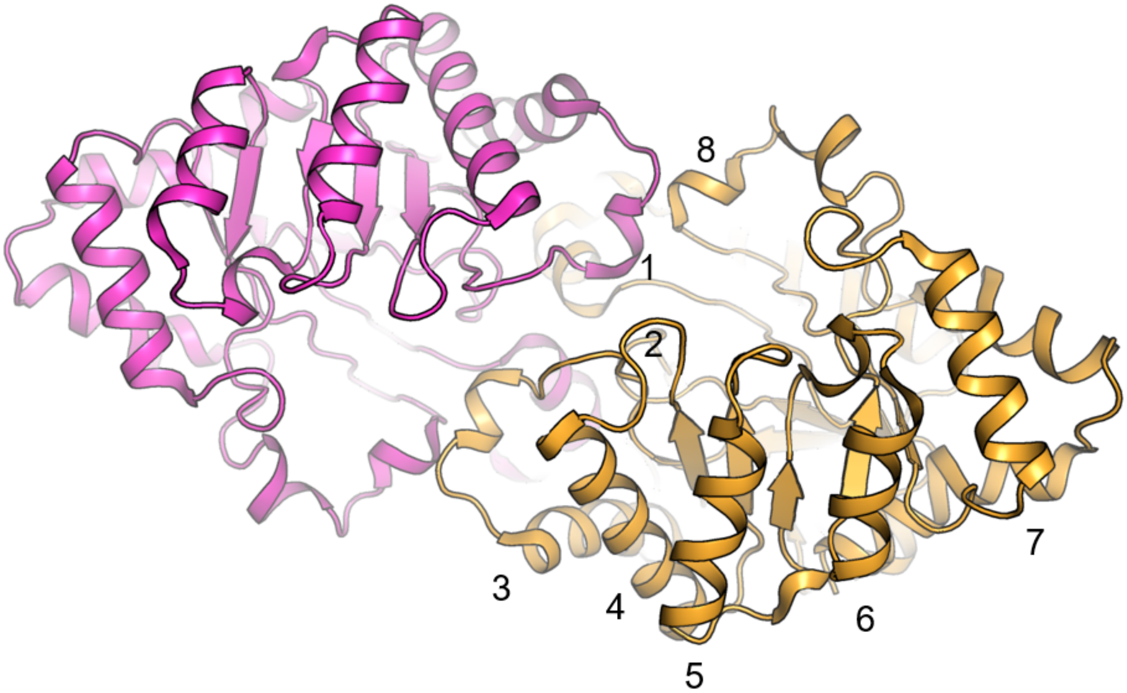

Supplementary Figure 1. PLL family enzymes are obligatory homodimers. The dimerization interface comprises β-α units 1-3, and 8.

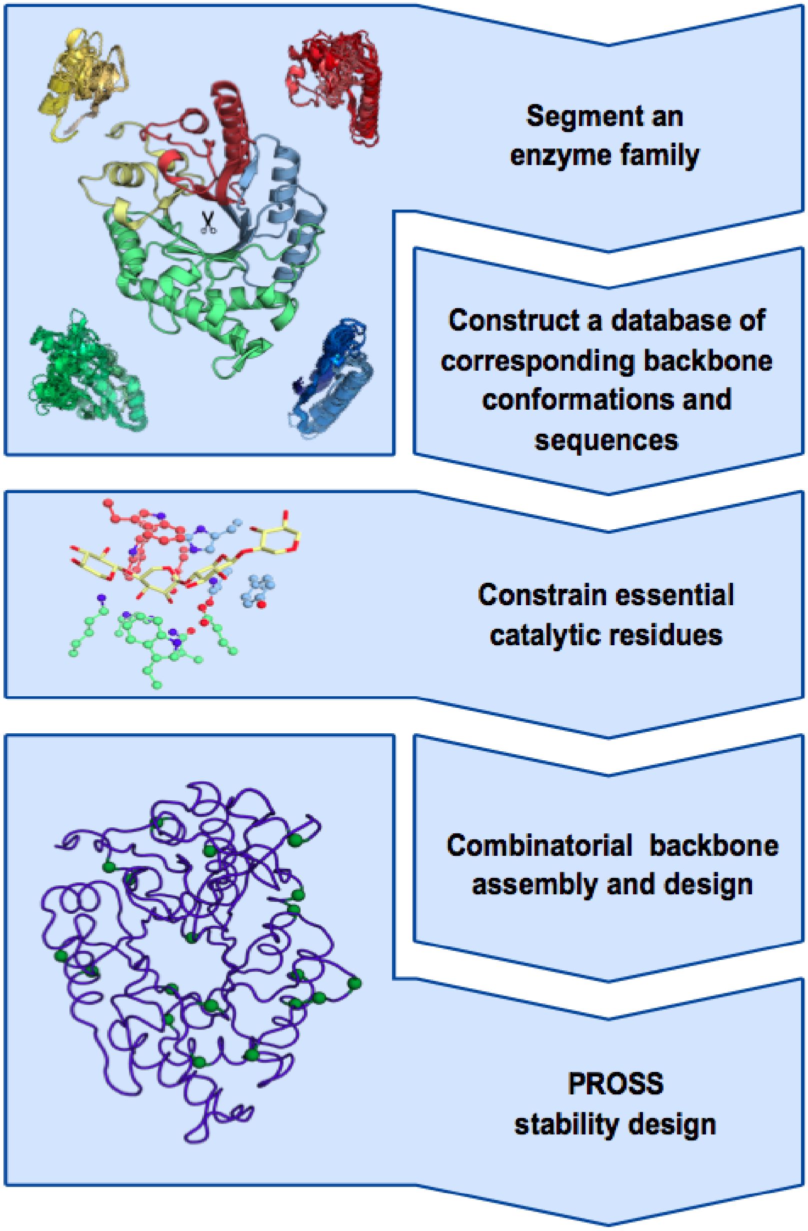

Supplementary Figure 2. Schematic representation of the algorithm.

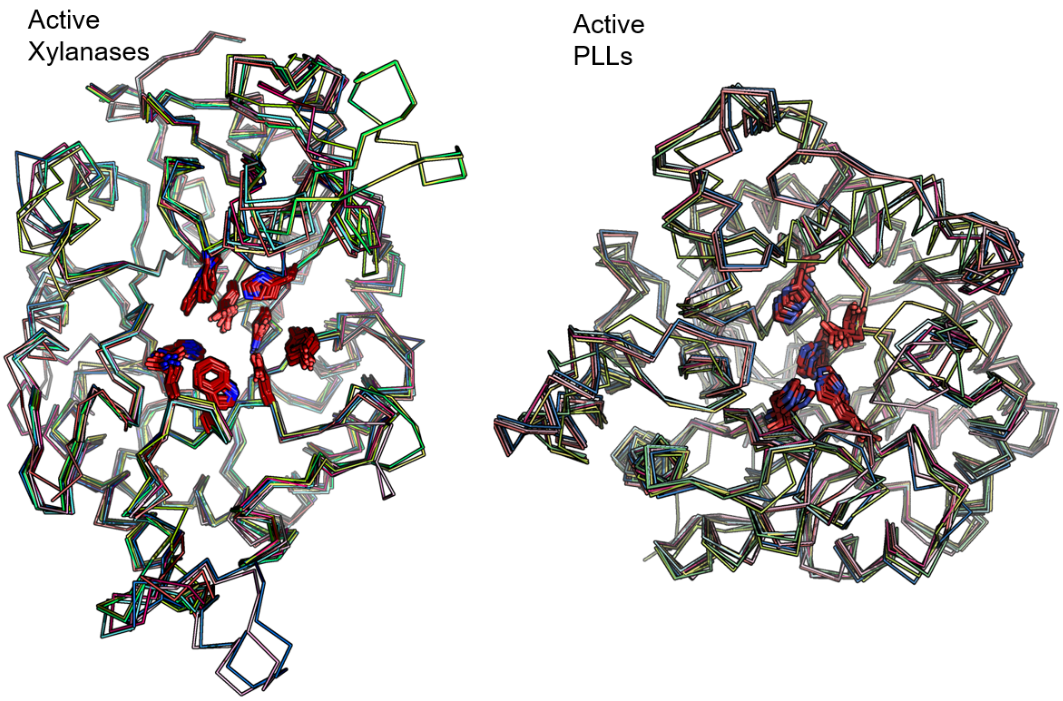

Supplementary Figure 3. Structural alignment of models of the active designs. The design models show high diversity in the backbone conformation relative to one another, while conserving active-site residues.

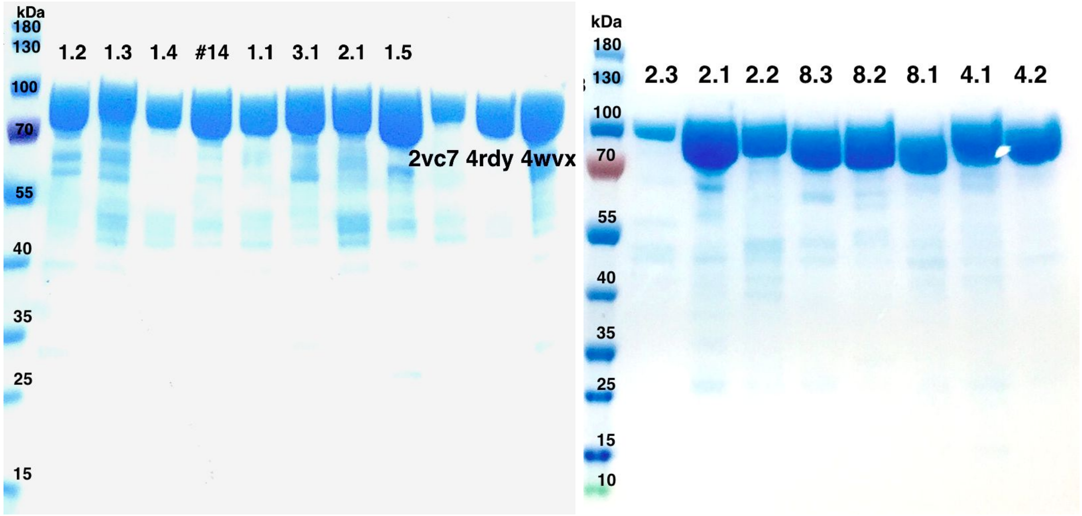

Supplementary Figure 4. SDS-PAGE analysis of eluted PLL designs (left) and GH10 designs (right), following purification with amylose resin shows high expression yields for the designs.

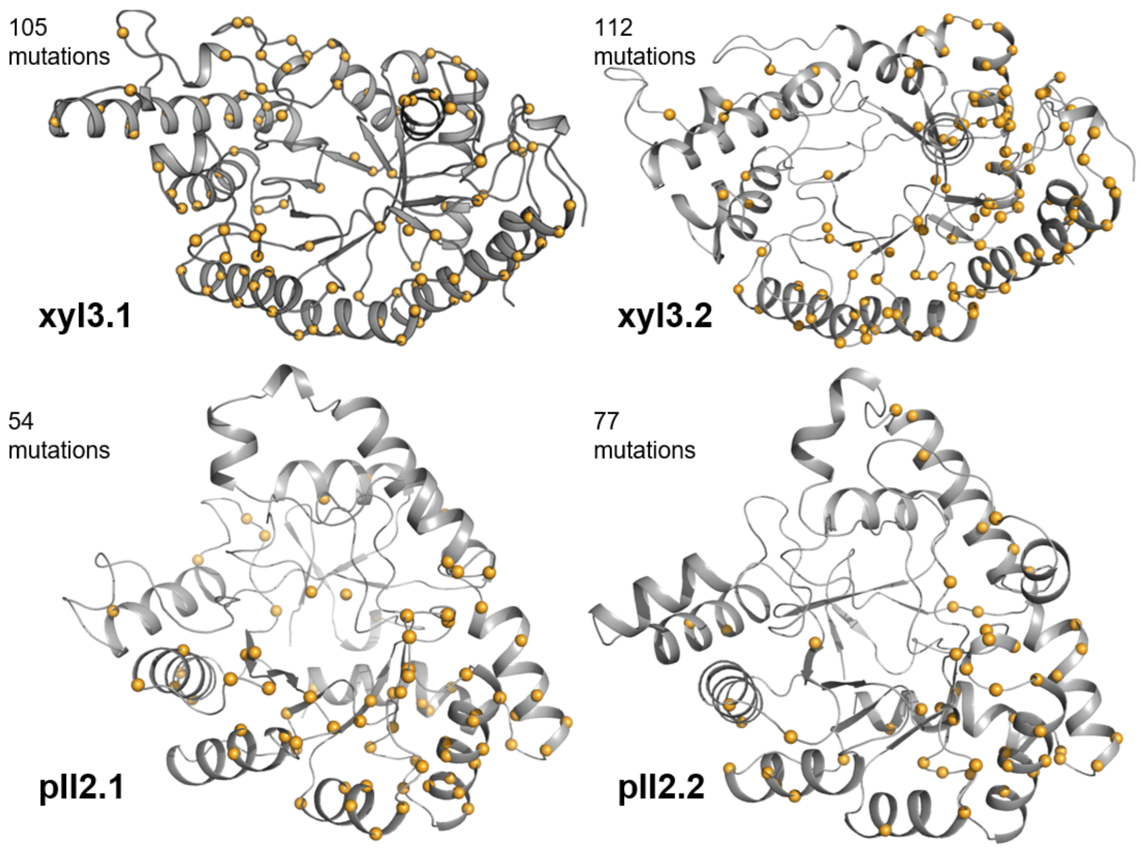

Supplementary Figure 5. Mutations (orange spheres) in the most active designs relative to the closest natural homologue.

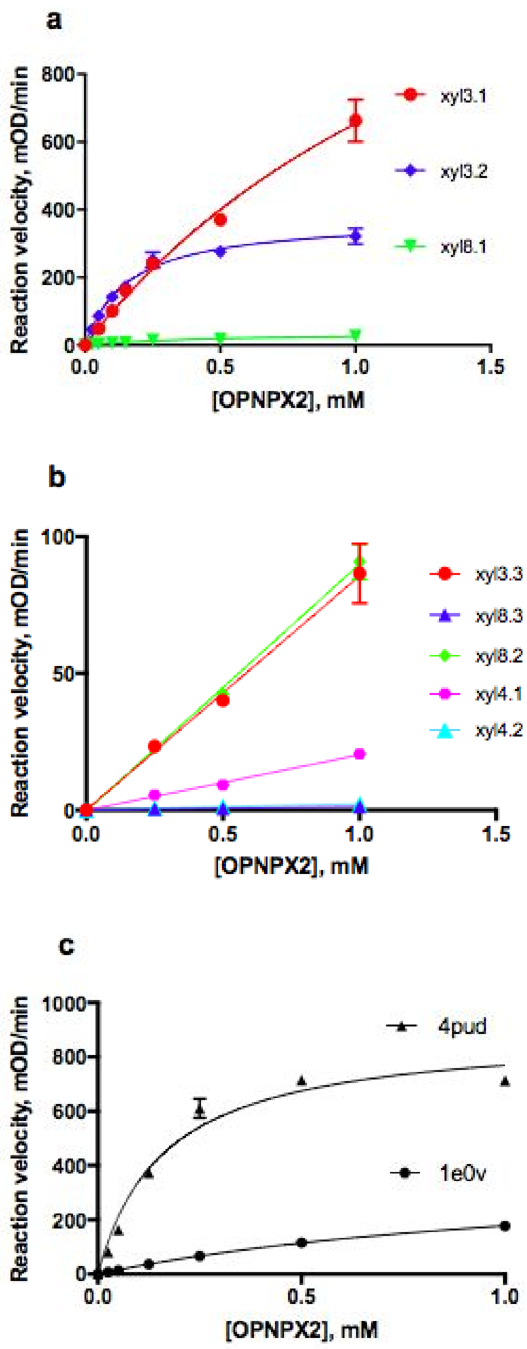

Supplementary Figure 6. Michaelis Menten plots of GH10 designs (a, b) and natural GH10 enzymes (GH10 xylanase from *G. stearothermophilus*, PDB ID 4PUD, and GH10 xylanase from *S. lividans*, PDB ID 1E0V) (c) with 4-nitrophenyl β-xylobioside (OPNPX$_2$). Protein concentrations are: xyl3.1 - 0.6875 µM, xyl3.2 - 2.925 µM, xyl3.3 - 5.625 µM, xyl4.1 - 3.5 µM, xyl4.2 - 11.8 µM, xyl8.3 - 13.2 µM, xyl8.2 - 7.65 µM, xyl8.1 - 2.95 µM, 1e0v - 0.32 µM, 4pud - 0.8725 µM.Data are the means ± standard deviation of duplicate reactions.

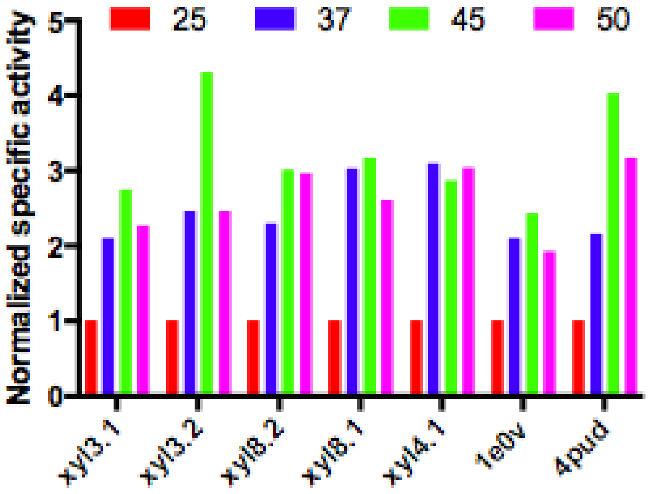

Supplementary Figure 7. Temperature dependence of designed and natural GH10 enzymes. Shown is specific activity (μM product per min for mg protein) with 1 mM $PNPX_2$, normalized to that at 25°C. Data are the means ± standard deviation of duplicate reactions.

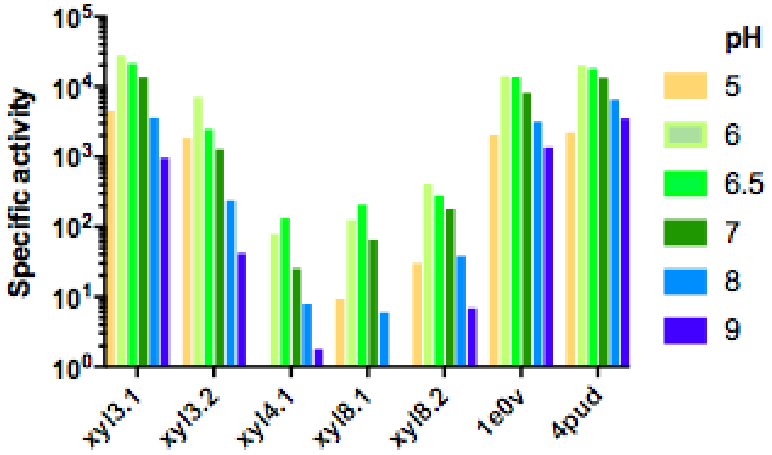

Supplementary Figure 8. pH dependence of natural and designed GH10 xylanase. Shown is specific activity (μM product per min for mg protein) with 1 mM $OPNPX_2$. Data are the means ± standard deviation of duplicate reactions.

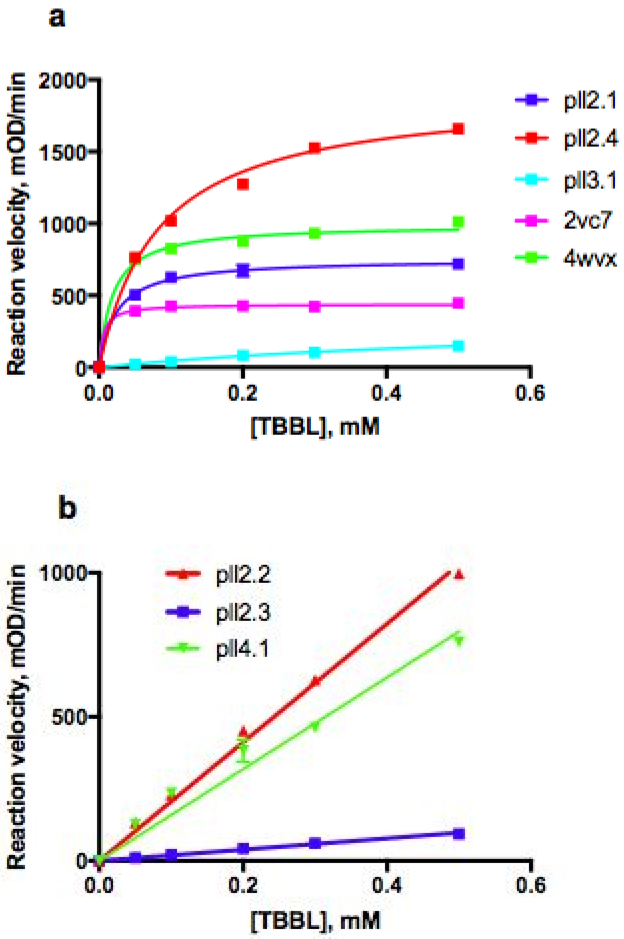

Supplementary Figure 9. Michaelis Menten curves of designed and natural PLLs with TBBL. Protein concentrations are: pll2.1 - 0.14 µM, pll2.2 - 0.14 µM, pll2.3 - 0.74 µM, pll2.4 - 1.15 µM, pll2.5 - 3.77 µM, pll3.1 - 1.84 µM, pll4.1 - 0.18 µM, 2vc7 - 0.83 µM, 4wvx - 1.61 µM. Data are the means ± standard deviation of duplicate reactions.

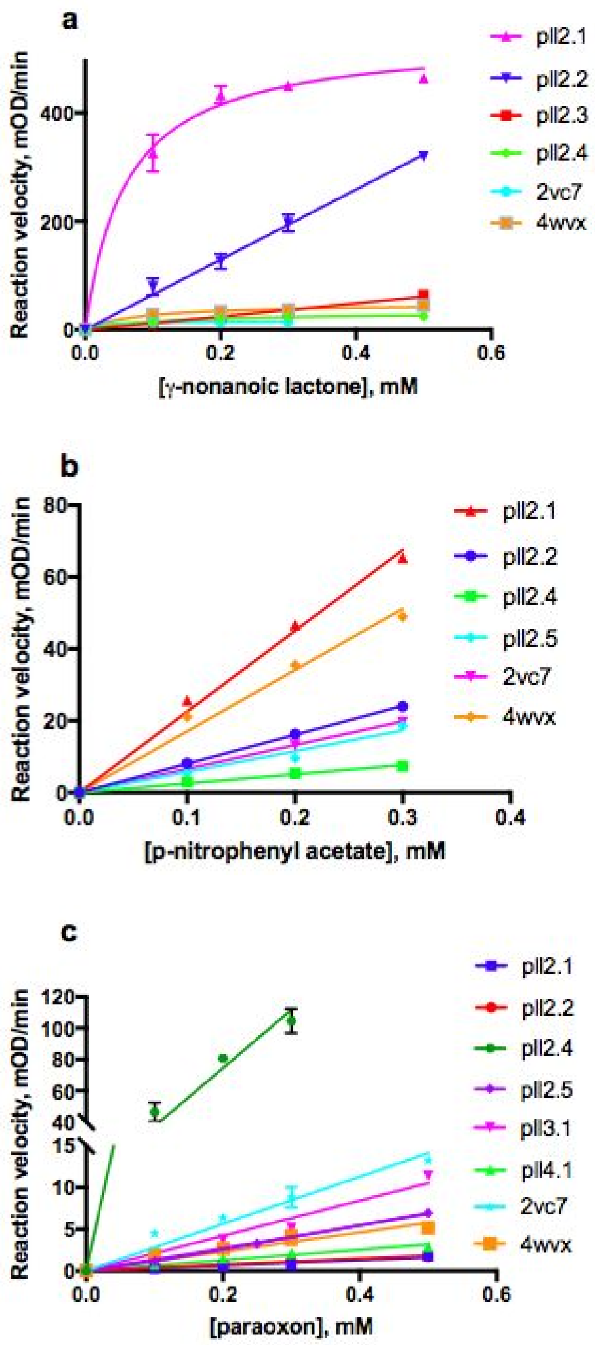

Supplementary Figure 10. (a) Catalytic efficiency of PLL designs and templates with γ-nonanoic lactone. Protein concentrations: pll2.1 - 2.31 μM, pll2.2 - 2.29 μM, pll2.4 - 1.65 μM. (b) Catalytic efficiency of designed and natural PLLs with p-nitrophenyl acetate. Protein concentrations: pll2.1 - 6.78 μM, pll2.2 - 7.08 μM, pll2.4 - 5.73 μM, pll2.5 - 8.875 μM, 2vc7 - 4.14 μM, 4wvx - 8.06 μM. (c) Catalytic efficiency of PLL designs and templates with paraoxon. Protein concentrations are as in (b). Data are the means ± standard deviation of duplicate reactions.

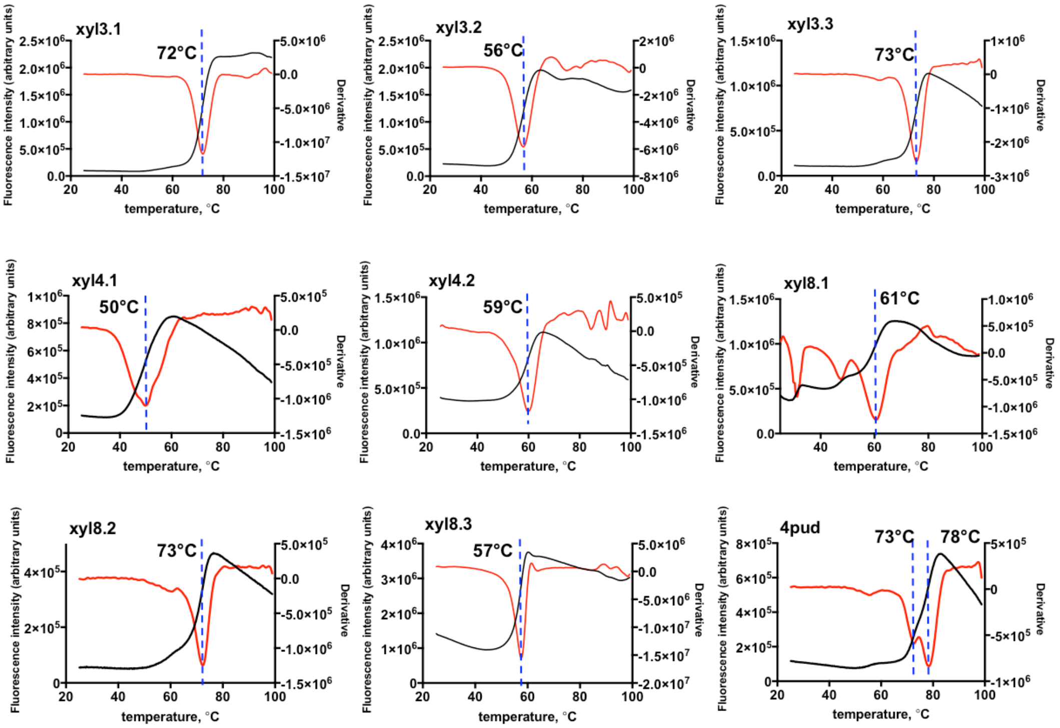

Supplementary Figure 11. Thermal denaturation of designed GH10s and a GH10 from *G. stearothermophilus* (PDB ID: 4PUD) by ThermoFluor using SYPRO Orange dye (black - fluorescence, red - derivative).

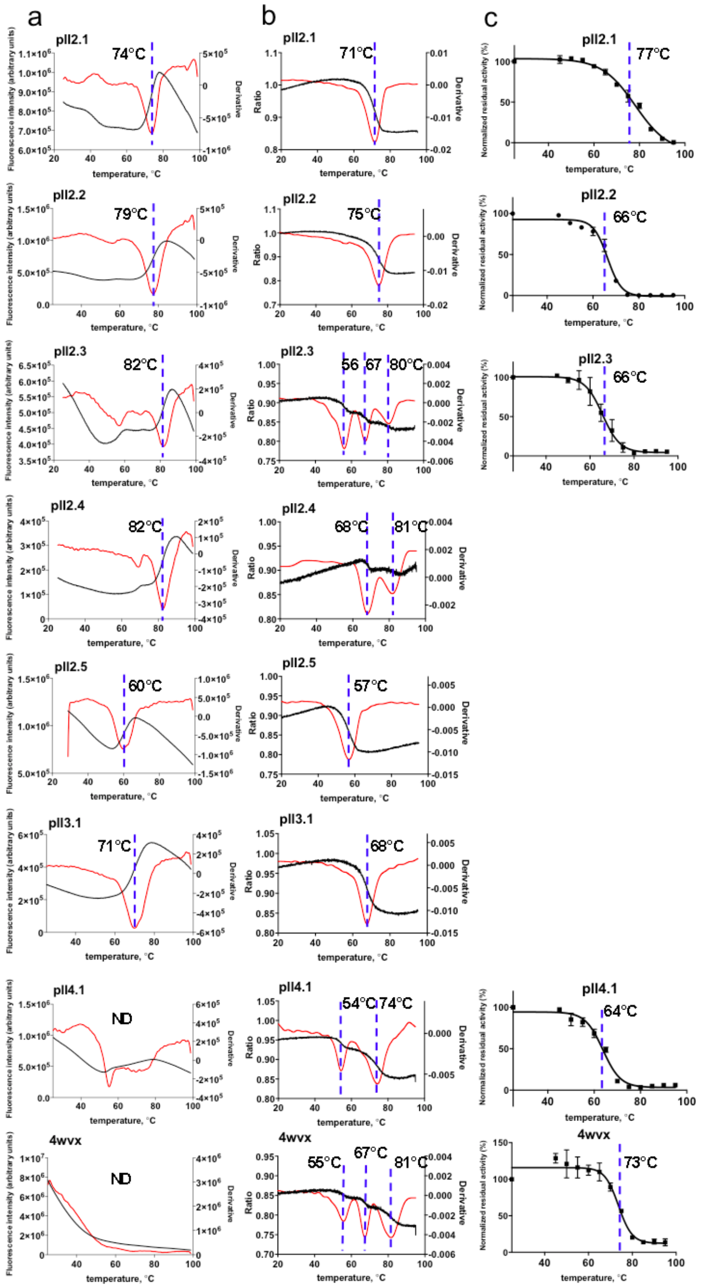

Supplementary Figure 12. (a) Thermal denaturation of designed and natural PLLs by ThermoFluor method with SYPRO Orange dye (black - fluorescence, red - derivative). Design pll4.1 did not show a clear melting transition. (b) Thermal denaturation of designed and natural PLLs by nanoDSF (black - ratio, red - derivative). Designs pll2.3, pll2.4, pll.4.1, and PLL from *G. kaustophilus* (PDB ID: 4WVX) exhibited multiple transitions, which can be attributed to inhomogeneity, such as monomers and dimers, present in solution. (c) Residual activity of PLL designs and natural PLL enzymes with 0.5 mM TBBL, following 0.5h incubation at different temperatures.

a

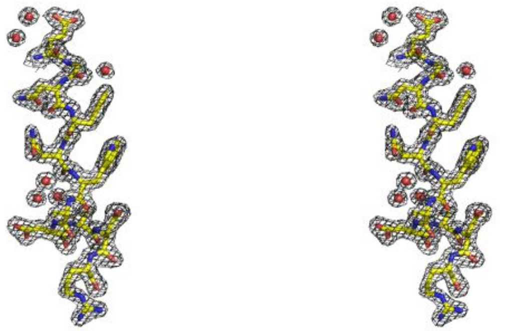

b

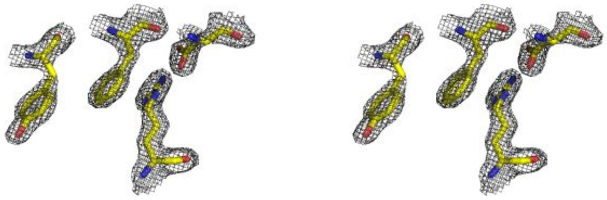

Supplementary Figure 13. Stereo electron density maps contoured at 1σ of (a) residues 70-80 region of the xy13.1 structure (PDB code 6FHF) and (b) Y68, F70, D74 and R141 of the xy18.3 structure (PDB code 6FHE). The grey mesh corresponds to the $2F_o$-F_c electron density map. Amino acids fitted to the $2F_o$-F_c map are shown as stick models, with carbons in yellow and water molecules are shown as red spheres.

Supplementary Tables

Supplementary Table 1. Extinction coefficients for monitoring the hydrolysis of various substrates (0.5cm optical length)[1]:

Substrate	ε, OD M^{-1}	Wavelength, nm
Paraoxon, p-nitrophenyl acetate (p-nitrophenol at pH 8.0)	11,230	405
4-nitrophenyl β-xylobioside (p-nitrophenol at pH 5.0)	890	405
4-nitrophenyl β-xylobioside (p-nitrophenol at pH 6.0	1,290	405
4-nitrophenyl β-xylobioside (p-nitrophenol at pH 6.5)	2,650	405
4-nitrophenyl β-xylobioside (p-nitrophenol at pH 7.0	5,850	405
4-nitrophenyl β-xylobioside (p-nitrophenol at pH 8.0	11,230	405
4-nitrophenyl β-xylobioside (p-nitrophenol at pH 9.0	12,330	405
TBBL (detection with Ellman's reagent, DTNB)	7,000	412
γ-nonanoic lactone (detection with m-cresol purple)	1,200	577

Supplementary Table 2. Catalytic efficiency of GH10 designs and wild-type GH10s with 4-nitrophenyl β-xylobioside ($PNPX_2$).

Xylanase	k_{cat}, s^{-1}	K_M, mM	k_{cat}/K_M, $s^{-1}M^{-1}$
xyl3.1	17±5	1.82±0.6	9417±311
xyl3.2	0.80±0.07	0.16±0.02	5060±280
xyl3.3			96±10
xyl4.1			36.3±0.1
xyl4.2			1.297±0.004
xyl8.1	0.084±0.006	0.54±0.05	156±4
xyl8.2			74±4
xyl8.3			0.61±0.01
Xylanase from *S. lividans* (PDB ID: 1E0V)	7.9±0.8	1.26±0.12	6722±30
Xylanase from *G. stearothermophilus* (PDB ID: 4PUD)	6.47±0.04	0.16±0.02	39,700±3,570

Supplementary Table 3. Catalytic efficiency of PLL designs with various substrates.

PLL	Metal ion	TBBL			γ-nonanoic lactone			para-oxon	p-nitro phenyl acetate
		k_{cat}, s^{-1}	K_{M}, mM	k_{cat}/K_{M}, $s^{-1}M^{-1}$	k_{cat}, s^{-1}	K_{M}, mM	k_{cat}/K_{M}, $s^{-1}M^{-1}$	k_{cat}/K_{M}, $s^{-1}M^{-1}$	k_{cat}/K_{M}, $s^{-1}M^{-1}$
pll2.1	Co^{2+}	12.8 ±0.3	0.023 ±0.004	556522 ±13810	5.52 ±0.25	0.06 ±0.01	92000 ±4250	0.78 ±0.07	55.4 ±1.6
	Zn^{2+}	2.09 ±0.05	0.065 ±0.005	31921 ±1746	0.9 ±0.1	0.11 ±0.01	8046 ±401	N.D.[(a)]	24.7 ±0.6
pll2.2	Co^{2+}	N.C.[(b]	N.C.	35000 ±780	N.C.	N.C.	12636 ±235	0.87 ±0.05	18.9 ±0.1
	Zn^{2+}	1.79 ±0.13	0.18 ±0.03	9944 ±173	N.C.	N.C	1720 ±83	N.D.	18.85 ±0.05
pll2.3	Co^{2+}	N.C.	N.C.	629 ±15	N.C.	N.C.	2270 ±99	N.D.	N.D.
	Zn^{2+}	N.C.	N.C.	275.5 ±4.9	N.C.	N.C.	N.D.	N.D.	N.D.
pll2.4	Co^{2+}	3.96 ±0.09	0.082 ±0.007	48290 ±1160	7.5 ±0.4	0.12 ±0.02	62500 ±3300	108 ±6	7.5 ±0.2
	Zn^{2+}	N.C.	N.C.	70.6 ±2.8	N.C.	N.C.	473 ±31	N.D.	N.D.
pll2.5	Zn^{2+}	N.C.	N.C.	7.3±0.3	N.C.	N.C.	N.D.	6.06 ±0.02	3.38 ±0.05
pll3.1	Co^{2+}	0.46 ±0.05	0.69 ±0.11	667 ±70			N.D.	3.8 ±0.2	N.D.
	Zn^{2+}	N.C.	N.C.	4.02 ±0.06	N.C.	N.C.	N.D.	N.D.	N.D.
pll4.1	Co^{2+}	N.C.	N.C.	21060 ±1100			N.D.	1.16 ±0.06	N.D.
	Zn^{2+}	0.57 ±0.05	0.35 ±0.05	1655 ±106	N.C.	N.C.	170±6	N.D.	N.D.
PLL from *S. solfatari*	Co^{2+}	1.26 ±0.03	0.006 ±0.002	210000 ±4447	0.26 ±0.01	0.017 ±0.007	15294 ±620	11.4 ±0.7	26.6 ±0.6
	Zn^{2+}	2.63	0.0145	181720	0.37	0.009	40736	N.D.	89

cus (PDB ID: 2VC7)		±0.02	±0.0005	±3470	±0.01	±0.001	±1203		±3
PLL from *G. kaustophilus* (4WVX)	Co^{2+}	1.47 ±0.04	0.019 ±0.004	77370 ±2106	0.41 ±0.02	0.078 ±0.016	5256 ±290	2.4 ±0.2	35.3 ±1.5
	Zn^{2+}	0.137 ±0.006	0.015 ±0.005	9356 ±1182	0.065 ±0.001	0.035 ±0.005	1866 ±222	N.D.	84 ±8

(a) No activity was detected.

(b) K_M was too high, and catalytic efficiency was calculated at linear regime of the Michaelis-Menten model.

Supplementary Table 4. Data collection and refinement statistics for the designed GH10 enzymes xyl3.1 and xyl8.3

	xyl8.3	xyl3.1
**Data Collection**		
PDB code	6FHE	6FHF
Space group	$P4_12_12$	*H3*
Cell dimensions:		
a,b,c (Å)	51.02, 51.02, 296.38	128.97, 128.97, 51.71
α, β, γ (°)	90, 90, 90	90, 90, 120
No. of copies in a.u.	1	1
Resolution (Å)	38.67-1.93	46.9-1.85
Upper resolution shell (Å)	1.99-1.93	1.85-1.95
Unique reflections	30,537(3,018)	27,384 (4,010)
Completeness (%)<br>Multiplicity	98.7(100.0)<br>6.3(6.8)	100.0(100.0)<br>5.2(5.0)
Average I/σ(I)	8.2(1.1)	12.7(2.7)
Wilson B-factor (Å^2)	39.11	24.58
Rsym (I) (%)	3.9(64.6)	7.3(52.8)
**Refinement**		
Resolution range (Å)	38.67-1.93	37.2-1.85
No. of reflections (I/σ(I) > 0)	30,500	27,375

No. of reflections in test set	1,539	2,747
R-working (%) / R-free (%)	24.2/27.3	18.4/22.6
No. of protein atoms	2,540	2,936
No. of water molecules	71	90
Overall average B factor (Å^2)	44.36	26.14
B factor for protein(Å^2)	44.36	26.13
B factor for water(Å^2)	30.00	25.74
Root mean square deviations:		
- bond length (Å)	0.008	0.009
- bond angle (°)	1.56	0.98
CC1/2^2	0.999(0.74)	0.875(0.632)
**Ramachandran Plot**		
Most favored (%)	96.1	97.8
Additionally allowed (%)	3.9	1.7
Disallowed (%)	0.0	0.5

* Values in parentheses refer to the data of the corresponding upper resolution shell

Supplementary Table 5. PDB structures from which active designs were assembled.

Design name	Backbone templates	
	β-α unit #	PDB ID
xyl3.1	1,5-6	4PUE
	2-4	2DEP
	7-8	2F8Q
xyl3.2	1,5-6	4PUE
	2-4	3W28
	7-8	2F8Q
xyl3.3	1,5-6	4PUE
	2-4	1UQZ
	7-8	2F8Q
xyl4.1	1	1CLX
	2-4	3MUI
	5-6	1US3
	7-8	1R86
xyl4.2	1	4QDM
	2-4	3MS8
	5-6	1VBR
	7-8	1R86
xyl8.1	1	4PMV
	2	5AY7
	3	1GOK
	4	3WUB
	5	1VBR
	6	1E0X
	7	4QCE
	8	3W27
xyl8.2	1	2DEP
	2	3W24
	3	1R87
	4	3NIY
	5	1CLX
	6	3W24
	7	1N82
	8	4W8L
xyl8.3	1	3W28
	2	5AY7
	3	1R87
	4	3W26
	5	1OD8
	6	3MSD
	7	3W25
	8	4PMU
pll2.1	7	5CH9
	1-6,8	2VC5

pll2.2	7	2VC5
	1-6,8	4WVX
pll2.3	7	3RHG
	1-6,8	4WVX
pll2.4	7	3OJG
	1-6,8	2VC5
pll2.5	7	4KF1
	1-6,8	1JGM
pll3.1	4	4KF1
	7	4H9X
	1-3,5-6, 8	2VC5
pll4.1	4	3GU2
	5	4XAY
	6	3RHG
	1-3,7-8	4WVX

Supplementary Table 6. Primers used for cloning of PLL and GH10 designs.

Primer name	5’-3’ sequence
EcoRI_fo	AGGATTTCAGAATTCATCACCAACAGCGGCGATCGGATC
PstI_bc	TGCCAAGCTTGCCTGCAGTCAGCTCGCGCGCAGGGTCGGGCTCAG
pETMBPH_seq_fo	TCCGCGGGTGAAAACCTGTACTTCCAGGGT
T7_seq_bc	CCCGTTTAGAGGCCCCAAGGG

Supplementary Note 1.

Amino acid sequences of the active GH10 xylanase designs.

Genes encoding these designs are available from AddGene, see Methods.

> xyl3.1

KPHISALNAPSLAQRYKDYFYIGAAVEPYQTTKEKDAKMLQRHFNMIVAENAMKPAALEPTEGNF
QWADADRIVQFAKENGMELRFHTLVWHNQTPDWFFLDREGKPMVEETDPQKREENRELLLQRL
ENHIRAVVLRYKDDIKNWDVVNEVVEPNDPGGMRNSPWYQITGTEYIEVAFRAAREAGGEDIKL
YMNDYNTEQEPKREYIYRLIKKLLEKGVPIDGVGHQAHVTIDRPPVDEIKKTIQRFADLGLDNQITE
LDVSLYGWPPRPAYPTYDAIPEERFQAQADRYRQLFELFEELKDHISAVTFWGIADNHTWLDDRA
REYNDGVGKDAPFVFDPNYRVKPAYWAIINHK

> xyl3.2

KPHISALNAPSLAERYKNYFYIGAAVEPDQTTKEKNAKMLRRHFDMIVAENAMKPEALEPTEGNF
TFDNANKIVDFAIAHNMKMRGHTLLWHNQIPEWFFRDPSDPSKPASRDLLLQRLRDHITTVLDHF
KTKYGSQNPIIGWDVVNEVLDDNGNLRNSKFLQIIGPDYIEKAFRFAHEADPSAKLFMNDYNTEQE
PKRQGIYELIKKLKERGAPIDGVGMQAHVTIDWPPVDEIKKAIQMFAALGLEVQITELDVSLYGWP
PRPAYPTYDAIPEERFQAQADRYRQLFELFEELKDKISAVTFWGIADNHTWLDDRAREYNNGVGK
DAPFVFDPNYRVKPAYWAIINHK

> xyl3.3

KPHISALNAPSLAQRFKDYFRVGAAVHPSQTTKEKDAKMLRRHFNAITPENSMKWGVLQDAQGR
WNWRDADAFVNFGEKHNMNIRGHTLVWHAQIPDWVFKNPDGSYISKEALLKRMENHITTLVGR
YKGKVHAWDVVNEAVGEDGRMRDSHWYRIVGDDFIRDAFRYAHEADPDAKLFYNDYNTEVEG
KREAIYRLIEKLKKKGVPIDGVGIQAHVTIDRPPVDEIKKTIQRFADLGLEVQITELDVSLYGWPPRP
AYPTYDAIPEERFQAQADRYRQLFELFEEMKDKLSSVTFWGIADNHTWLDDRAREYNNGVGKDA
PFVFDPNYRVKPAYWAIIDHK

> xyl4.1

TIQNDIPSLYEVFKDYFPIGVAVAATGGNADLFTSSAAQDMVRQHFNQITAENEMKPEHLQPEEGR
FTFEAADRMVDFAQKHGMKVRGHTLVWHNQTPDWMFKDDNGQPVSREQLLERMRNHIKTVVG
RYKGKVYCWDVVNEAISDNGSDCLRDSRWRQIIGDDFMEEAFRYAHEADPDAKLFMNDYNIESN
GAKTDAMYNLVKEFLARGVPIHGVGFQGHIGLDWPSLDEIEEAIQRIADLGLDVAITELDVSMYG
WPPRAYPTYDAIPEQKFLDQAERYRELMQAFRKYADHISAVTFWGIADNHTWLDSRADVYYDSN
GNVVTDPNAPYARVEKGKGKDAPFVFGPDYNVKPAFWAIV

> xyl4.2

TIQTDIPDLYEVFKDYFPIGAAVEPRQLNGPEGKLIKKHFNSLTAENEMKPERLEPREGVFNFENAD
RIVDFAQKNNMKVRGHTLVWHNQTPDWVFKDDNGQPVSKEELLERMRNHIKTVVGRYKGKIYA

WDVVNEAVADEGTEVLRPSKWREIIGEDYIEEAFRYAHEADPDAKLFMNDYNIEGINAKSDALYN
LVKRLKERGVPVDGIGFQMHININGVDMDNFRKAIERFASLGLEVAITELDVSMYGWPPRAYPTY
DAIPEQKFLDQAERYKELMNAFREYADHITSVTFWGIADNHTWLDSRADVYYDSNGNVVTDPNA
PYARVEKGKGKDAPFVFGPDYNVKPAYWAVV

> xyl8.3

TIQNDIPDLYSVFKDYFPIGVAVDPSRLNDTDPHAQLTAKHFNMLTAENAMKPESLEPEEGRYNFE
DADRIVAFAEKHGMKMRGHTLVWHQQVPDWFFLDENGNPMVDETDPKNREANREELRQRMEN
HIKTVAGRYKGKIYAWDVVNEVFNDDGTLRNSAWYQIIGPDYIEEALRAAHEADPNAKLFINDYN
IENWSHAKTQAMYNMVRDFKERGVPVDGVGMQGHISLYYPSLEEIEKALKAFAALGVEIMITELD
VNTQGDVSPDALQEQAERMRDLFELFKKHSDKITGVTFWGVADDQSWKNNFPVPGRTNAPLLFD
RNYQPKPAFWAIV

> xyl8.2

TIQNDIPDLYSVFKDYFPIGAAVEPSQLSGPQAKLIQKHFNMLVAANAMKPESLQPTEGNFTFDNA
DKIVEFAIANNMKMRGHTLVWHNQVPDWFFQDPTDPSKPATREQLRERMREHIRTVLDHFRDKF
GSNNPIIGWDVVNEPLSDNGTYRPSPWYQIIGPDYIADAFRWAHAADPSAKLYLNDYGIEGNGAK
SDAMYNLVKDLQDRGVPIHGIGMQGHTHINSNPEWIEQAIERFASLGVEVHITELDVSMFAWDDK
RTDVTEPTEEMLERQAERYRELFEIFRKHSDVITSVTFWGVADGHSWLNNFPVKGRTDYPLLFDR
NYQPKKAFWAIV

> xyl8.1

TIQKDIPDLYKVFKDYFPIGTAVNTDIVEGRDAEAAALVRKHFNMLTAENAMKWMYLEPREGKF
DFEDADKIVNFAKKHGMKIRGHTLVWHSQLPSWVSNITDKNTLRKVMRNHITTVMGHYKGKIYG
WDVVNEALNDDGTYRQSVFYQVMGPDYIAEALRAAHEADPDAKLYINDYNIEGINAKSNALYEL
VRKLIERGVPIDGVGFQAHFSGGSPLCSNFRETIKRFAALGVDVQITELDMSIYGWPPRPAYETYDA
IPEEKFQQQAERMKKLFEMFKENSDYVTGVTFWGVRDADSWLGKGNAPLLFDSNGQPKRAFWA
VV

Amino acid sequences of the active PLL designs.

> pll2.4

MRIPLVGKDPIESKDIGFTLIHEHLRVFSEAVRQQWPHLYNEDEEFRNAVNEVKRAKAYGVDTIVD
PTVMGLGRDIRFMEKVAKATGINLIAGTGIYTYTDLPFYFLNRSIDEIADLFIHDITEGIQGTGNKAG
FVKCAADEPGITPDVEKVIRAAARAHKETGVPIITHSNPHHNTGLEQQRILEEEGVDPSRILIGHLGD
TTNINYIKKIADHGSFVAFDRFGIQGMMGAPTDEERLAALKALLDDGLADRIMISHDYCCTIDWGT
AKPEYKPKLAPRWSITLIFEDVIPALKENGVTEEQIRQIFVENPRRFFS

> pll2.1

VRISIAGGNEIDPGDMGLTLFHEHLRLITEVVRWNWPHLYNEDEELKRAIDAVRAAKKYGVKTIV
DCTVAGIGCDVRFVEKVAEATGVNIIMGTGFYTYTEIPFYFKNRGIDQLVDAFVHDITEGIQGTGV
RAGFVKCAIDHNGLTPDVEMVIRAAARAHRRTGVPIITHSHAGTKSGLEQIRIFKEEGVDLNRVVIG
HSGDTTDVDYLEQIARQGAFIAFDRFGLQGMVGAPTDEERIRSLKAMLDRGYADRIMLSHDYCPTI
DWYPPEVVRSAAPNWTMTHIFEHVIPALREAGVTEEQIDTILVDNPRRLFEG

> pll4.1

ETVNTVTGPVPVEQLGKTLIHEHFLFGYPGFQGDVTRGTFDEDEALERAIAEAEKMKAHGVKTVV
DPTPNDCGRDPRFLRKVAEATGLNIICATGYYYEGEGAPPYWKFRAALGTAEEEIYDMFVREITEG
IADTGIRAGVIKVASSKDGITPYEEKFFRAAARAHRETGAPIITHTHGWNRNGLEQADIFAEEGVDP
SKVVIGHMDGSGDDLDYQKEVADQGVYIAFDRFGIQGMVGAPTDEERIAALLALLKDGYADRIM
LSHDTVNVWLGRPFVLPEALQEMMKNWHVGHLFDNVIPMLKEHGVTDEQIDQMFVENPARLFS

> pll3.1

MRIPLVGKDPIESGDIGFTLIHEHLRVFSEAVRQQWPHLYNEDEELRNAVNEVRKAMAHGVDTIV
DPTVMGLGRDIRFMQKVARETGINLVAGTGIYIYTDLPFYFLNRSIDEIADLFIRDIEEGIDGTGVRA
GFVKVASGEPGITPDVEKVIRAAARAHKETGAPIITHSNPHNRTGLEQLRILAEEGVDPRRILIGHM
DDTTDIDYIKEIADHGAFIGFDRFGIQGMVGCPTDDERIDTLKALLDDGYADKIMISHDYCCTIDW
GTARPEYKPKLAPRWSMTLIFDDVIPALKKNGVDDETINQIFVENPARFFS

> pll2.5

GDRINTVRGPITISEAGFTLTHEHICGSSAGFLRAWPEFFGSRKALAEKAVRGLRRARAAGVRTIVD
VSTFDMGRDVDLLAEVSRAADVHIVAATGLWFDPPLSMRLRSVEELTQFFLREIQYGIEDTGIRAG
IIKVATTGKATPFQELVLRAAARAALETGVPVTTHTNASQRDGEQQADIFESEGLPPSRVLIGHCGD
TDDLDYLRALADRGYYIGFDTIGKNNYQPDERRIEMIAEMVKRGYADRILISHDYLFGFSSYVTNI
MDVMDRINPDGMAHLPDRVIPALREAGVSDEQIRQMTVENPARFLSPQLRAS

> pll2.2

EMVETVCGPVPVEQLGKTLIHEHFLFGYPGFQGDVTRGTFNEEEALRVAVEEAERMKAHGVRTV
VDPTPNDCGRNPAFLRRVAEETGLNIICATGYYYEGEGAPPYFQFRRLLGTAEDDIYEMFVAELTE
GIADTGIKAGVIKLASSKGGITEYEEMFFRAAARAQRETGAVIITHTQEGTMGPEQARLLKEEGAD
PDKIVIGHMGDNDDLDYHRWTLAYGVYIGFDGFGRDIYLPWERRVELIAALIRDGYADRILLSHDS
VNVWLGRPFTWPEPFAEMMKNWHVEHLFRTVIPALKERGVTDEDIEQMFVGNPARLFS

> pll2.3

EMVNTVTGPVPVDQLGKTLIHEHFLFGYPGFQGDVTRGTFDEEEALRRAVEEAERMKAHGVRTV
VDPTPNDCGRNPAFLRRVAEETGLNIICATGYYYEGEGAPPYFKFRRALGTAEDDIYDMFVAEITE
GIADTGIKAGVIKCASSKGGITPYEEMFFRAAARAQRETGAPIITHTTAGTMGPEQARLLKEHGAD
PSRIVIGHMGDNTDPDYHRKTLDYGVYVAFDMIGLDISFPGEGQAPSDEETADMIAALIDDGYADR
IMLSHDSVNVWLGRPFVWPPPMQEMVKNWHVEHLFEKFIPRLKERGVSDEDIEQMLIGNPRRLFS

Supplementary References

1. Khersonsky, O. & Tawfik, D. S. Structure-reactivity studies of serum paraoxonase PON1 suggest that its native activity is lactonase. *Biochemistry* **44,** 6371–6382 (2005).
2. Karplus, P. A. & Diederichs, K. Assessing and maximizing data quality in macromolecular crystallography. *Curr. Opin. Struct. Biol.* **34,** 60–68 (2015).
