# Supplementary material for: Highly active enzymes by automated combinatorial backbone assembly and sequence design
Source: Nat Commun. 2018 Jul 17;9:2780. doi: 10.1038/s41467-018-05205-5 (PMC6050298; doi:10.1038/s41467-018-05205-5)
Supplement: Supplementary file 3 — Description of Additional Supplementary Files [file 41467_2018_5205_MOESM3_ESM.docx]

**Description of Additional Supplementary Files**

**File Name:** Supplementary Movie 1

**Description:** Video describing the design process

**File Name:** Supplementary Data 1

**Description:** README file for conformations db preparation.

Explanation on how to run conformation db creation and PSSM creation

**File Name:** Supplementary Data 2

**Description:** An example of a rotamer database file - not used during design

**File Name:** Supplementary Data 3

**Description:** Sequence alignment file used to create a conformation specific PSSM

**File Name:** Supplementary Data 4

**Description:** File used to generate the conformation database for unit 4

**File Name:** Supplementary Data 5

**Description:** Source conformation used to generate conformation db

**File Name:** Supplementary Data 6

**Description:** PSSM file used during design and creation of conformation db file

**File Name:** Supplementary Data 7

**Description:** Python script for aligning segments between source pdb and template pdb

**File Name:** Supplementary Data 8

**Description:** Parameters passed to create MSA file

**File Name:** Supplementary Data 9

**Description:** Parameters used to create conformation db

**File Name:** Supplementary Data 10

**Description:** XML for generating MSA using Rosetta

**File Name:** Supplementary Data 11

**Description:** XML for generating conformation db

**File Name:** Supplementary Data 12

**Description:** Instructions on how to run the enzyme design protocol using the enclosed files

**File Name:** Supplementary Data 13

**Description:** βα unit 4 conformation database file
Text file where each line is an entry containing the ψ, φ, and ω dihedral angles of every segment conformation sampled from the natural enzyme family

**File Name:** Supplementary Data 14

**Description:** βα unit 5 conformation database file
Text file where each line is an entry containing the ψ, φ, and ω dihedral angles of every segment conformation sampled from the natural enzyme family

**File Name:** Supplementary Data 15

**Description:** βα unit 6 conformation database file
Text file where each line is an entry containing the ψ, φ, and ω dihedral angles of every segment conformation sampled from the natural enzyme family

**File Name:** Supplementary Data 16

**Description:** βα unit 7 conformation database file
Text file where each line is an entry containing the ψ, φ, and ω dihedral angles of every segment conformation sampled from the natural enzyme family

**File Name:** Supplementary Data 17

**Description:** This PDB file was used during the computational design process to constraint the catalytic residues of PLL family enzymes

**File Name:** Supplementary Data 18

**Description:** This file is an example input file used for the conformation sampling stage

**File Name:** Supplementary Data 19

**Description:** This PDB file was used during the computational design process to

constraint the catalytic residues of PLL family enzymes

**File Name:** Supplementary Data 20

**Description:** This PDB file was used during the computational design process to

constraint the catalytic residues of PLL family enzymes

**File Name:** Supplementary Data 21

**Description:** Parameter files:

*Flags_pssm -* Parameter file mapping from PDB ID to path of corresponding PSSM file. *Flags_pross1* - First parameter file used during the stabilization stage of the design protocol. *Flags_pross2 -* Second parameter file used during the stabilization stage of the design protocol.

*Flags_slice_in* - Parameter file used during conformation sampling stage

**File Name:** Supplementary Data 22

**Description:** PSSM file of the N-terminal segment of the template PDB 2VC5

**File Name:** Supplementary Data 23

**Description:** PSSM file of the C-terminal segment of the template PDB 2VC5

**File Name:** Supplementary Data 24

**Description:** Text file where each line has a PDB name entry and a PSSM file name

**File Name:** Supplementary Data 25

**Description:** Rosetta scripts XML files:

*Filterscan_auto_xsd.xml* - Part of the PROSS stabilization algorithm, calculates the resfile that is later used during design.

*Design_auto_xsd.xml* - Part of the PROSS stabilization algorithm, uses the previously generated resfile to introduce stabilizing mutations on the protein. *Splice_constraints_xsd.xml -* XML file used to apply sequence constraints to the pose during design.

*Splice_in_xsd.xml* - XML file used during the conformation sampling stage of the protocol
