# Supplementary material for: Highly active enzymes by automated combinatorial backbone assembly and sequence design
Source: Nat Commun. 2018 Jul 17;9:2780. doi: 10.1038/s41467-018-05205-5 (PMC6050298; doi:10.1038/s41467-018-05205-5)
Supplement: Supplementary file 25 — Supplementary Data 21 [file 41467_2018_5205_MOESM25_ESM.pdf]

flags_pssm

```
-parser:script_vars blade4=# comma separated list of protein names and pssm paths, e.g. 1bf6A:pssm/blade4/1bf6A.pssm,1bf6B:pssm/blade4/1bf6B.pssm
-parser:script_vars blade5=# comma separated list of protein names and pssm paths, e.g. 1bf6A:pssm/blade5/1bf6A.pssm,1bf6B:pssm/blade5/1bf6B.pssm
-parser:script_vars blade6=# comma separated list of protein names and pssm paths, e.g. 1bf6A:pssm/blade6/1bf6A.pssm,1bf6B:pssm/blade6/1bf6B.pssm
-parser:script_vars blade7=# comma separated list of protein names and pssm paths, e.g. 1bf6A:pssm/blade7/1bf6A.pssm,1bf6B:pssm/blade7/1bf6B.pssm
```

flags_pross

```
-ex1
-ex2

-nodelay
-use_input_sc
-extrachi_cutoff 8
-ignore_unrecognized_res
-chemical:exclude_patches LowerDNA  UpperDNA Cterm_amidation SpecialRotamer VirtualBB ShoveBB VirtualDNAPhosphate VirtualNTerm CTermConnect sc_orbitals pro_hydroxylated_case1 pro_hydroxylated_case2 ser_phosphorylated thr_phosphorylated  tyr_phosphorylated tyr_sulfated lys_dimethylated lys_monomethylated  lys_trimethylated lys_acetylated glu_carboxylated cys_acetylated tyr_diiodinated N_acetylated C_methylamidated MethylatedProteinCterm
-jd2:ntrials 5
-overwrite
-out:file:fullatom
-pdb_comments true ## this is the mover reades the comments in the input pdb file. The comments contain the pdb segments comprising the input pdb



-out:path:pdb pdb/
-mute protocols.toolbox.task_operations.SeqprofConsensusOperation
-no_nstruct_label
#-mute devel.splice.Splice_constraints
#-mute devel.splice.SpliceSegment
#-mute protocols.rosetta_scripts.RosettaScriptsParser
#-mute core.pack.rotamer_set.SampleRotamersFromPDB_RotamerSetOperation
#-mute all
```

```
-unmute protocols.jd2.JobDistributor
-unmute devel.splice.util
-unmute protocols.simple_filters.ScoreTypeFilter

-parser:protocol filterscan_auto_xds.xml
@flags_pssm
```

flags_pross1

```
-ex1
-ex2

-nodelay
-use_input_sc
-extrachi_cutoff 8
-ignore_unrecognized_res
-chemical:exclude_patches LowerDNA  UpperDNA Cterm_amidation
SpecialRotamer VirtualBB ShoveBB VirtualDNAPhosphate VirtualNTerm
CTermConnect sc_orbitals pro_hydroxylated_case1 pro_hydroxylated_case2
ser_phosphorylated thr_phosphorylated  tyr_phosphorylated tyr_sulfated
lys_dimethylated lys_monomethylated  lys_trimethylated lys_acetylated
glu_carboxylated cys_acetylated tyr_diiodinated N_acetylated
C_methylamidated MethylatedProteinCterm
-jd2:ntrials 5
-overwrite
-out:file:fullatom
-pdb_comments true ## this is the mover reades the comments in the
input pdb file. The comments contain the pdb segments comprising the
input pdb



-out:path:pdb pdb/
-mute protocols.toolbox.task_operations.SeqprofConsensusOperation
-no_nstruct_label
#-mute devel.splice.Splice_constraints
#-mute devel.splice.SpliceSegment
#-mute protocols.rosetta_scripts.RosettaScriptsParser
#-mute core.pack.rotamer_set.SampleRotamersFromPDB_RotamerSetOperation
#-mute all
-unmute protocols.jd2.JobDistributor
-unmute devel.splice.util
-unmute protocols.simple_filters.ScoreTypeFilter
```

```
-parser:protocol filterscan_auto_xds.xml
@flags_pssm
```

flags_pross2

```
-ex1
-ex2

-nodelay
-use_input_sc
-extrachi_cutoff 8
-ignore_unrecognized_res
-chemical:exclude_patches LowerDNA  UpperDNA Cterm_amidation
SpecialRotamer VirtualBB ShoveBB VirtualDNAPhosphate VirtualNTerm
CTermConnect sc_orbitals pro_hydroxylated_case1 pro_hydroxylated_case2
ser_phosphorylated thr_phosphorylated  tyr_phosphorylated tyr_sulfated
lys_dimethylated lys_monomethylated  lys_trimethylated lys_acetylated
glu_carboxylated cys_acetylated tyr_diiodinated N_acetylated
C_methylamidated MethylatedProteinCterm
-jd2:ntrials 5
-overwrite
-out:file:fullatom
-pdb_comments true ## this is the mover reades the comments in the
input pdb file. The comments contain the pdb segments comprising the
input pdb




-out:path:pdb pdb/
-mute protocols.toolbox.task_operations.SeqprofConsensusOperation
-no_nstruct_label
#-mute devel.splice.Splice_constraints
#-mute devel.splice.SpliceSegment
#-mute protocols.rosetta_scripts.RosettaScriptsParser
#-mute core.pack.rotamer_set.SampleRotamersFromPDB_RotamerSetOperation
#-mute all
-unmute protocols.jd2.JobDistributor
-unmute devel.splice.util
-unmute protocols.simple_filters.ScoreTypeFilter

-parser:protocol design_auto_xsd.xml
@flags_pssm
```

flags_splice_in

```
-ex1
-ex2
-nodelay
-use_input_sc
-extrachi_cutoff 8
-ignore_unrecognized_res
-chemical:exclude_patches LowerDNA  UpperDNA Cterm_amidation
SpecialRotamer VirtualBB ShoveBB VirtualDNAPhosphate VirtualNTerm
CTermConnect sc_orbitals pro_hydroxylated_case1 pro_hydroxylated_case2
ser_phosphorylated thr_phosphorylated  tyr_phosphorylated tyr_sulfated
lys_dimethylated lys_monomethylated  lys_trimethylated lys_acetylated
glu_carboxylated cys_acetylated tyr_diiodinated N_acetylated
C_methylamidated MethylatedProteinCterm
-jd2:ntrials 5
-overwrite
-out:file:fullatom
-pdb_comments true ## this is the mover reades the comments in the
input pdb file. The comments contain the pdb segments comprising the
input pdb

-parser:protocol splice_in_xsd.xml
-parser:script_vars start_pdb=2vc5B_ppk.pdb

#-mute all
-out:path:pdb pdb/
@flags_pssm
-no_nstruct_label
```
