# Supplementary material for: Highly active enzymes by automated combinatorial backbone assembly and sequence design
Source: Nat Commun. 2018 Jul 17;9:2780. doi: 10.1038/s41467-018-05205-5 (PMC6050298; doi:10.1038/s41467-018-05205-5)
Supplement: Supplementary file 29 — Supplementary Data 25 [file 41467_2018_5205_MOESM29_ESM.pdf]

design_auto_xsd.xml

```
<ROSETTASCRIPTS>

	<SCOREFXNS>
		<ScoreFunction name="talaris_full" weights="ref2015">
			<Reweight scoretype="coordinate_constraint"
weight="0.4"/>
			<Reweight scoretype="res_type_constraint"
weight="0.4"/>
		</ScoreFunction>

		<ScoreFunction name="soft_rep_full"
weights="soft_rep">
			<Reweight scoretype="coordinate_constraint"
weight="0.4"/>
			<Reweight scoretype="res_type_constraint"
weight="0.4"/>
		</ScoreFunction>

		<ScoreFunction name="tal_no_pssm" weights="ref2015">
			<Reweight scoretype="coordinate_constraint"
weight="0.4"/>
		</ScoreFunction>

		<ScoreFunction name="talaris_pure" weights="ref2015"/>
	</SCOREFXNS>

		<TASKOPERATIONS>
				<RestrictIdentitiesAtAlignedPositions
name="RIAAP_design" source_pdb="4zstA.pdb.gz"
resnums="267,196,167,23,21,135" keep_aas="HDKE"
design_only_target_residues="1"/>keep cat arg
				<RestrictIdentitiesAtAlignedPositions
name="RIAAP_design_glu" source_pdb="2vc5B_ppk.pdb.gz" resnums="166"
keep_aas="HDKE" design_only_target_residues="1"/>
		</TASKOPERATIONS>
		<RESIDUE_SELECTORS>
			<Task name="RIAAP_design" packable="1"
designable="1" task_operations="RIAAP_design"/>RIAAP_design_glu" />
			<Not name="No_RIAAP_design"
selector="RIAAP_design"/>
		</RESIDUE_SELECTORS>

	<TASKOPERATIONS>



		<ProteinInterfaceDesign name="no_repack_ligand"
repack_chain2="0" />
```

```
                <RestrictIdentitiesAtAlignedPositions
name="no_repack_cat_Res" source_pdb="4zstA.pdb.gz"
prevent_repacking="1" resnums="267,196,167,23,21,135,134,136"
keep_aas="HDKE"/>keep cat arg
        <RestrictIdentitiesAtAlignedPositions name="no_repack_cat_glu"
source_pdb="2vc5B_ppk.pdb.gz" prevent_repacking="1" resnums="166"
keep_aas="HDKE"/>

                <OperateOnResidueSubset name="No_RIAAP_design"
selector="No_RIAAP_design" >
                   <RestrictToRepackingRLT/>
                </OperateOnResidueSubset>

                <InitializeFromCommandline name="init"/>
                <ReadResfile name="read_resfile" filename="resfile/
resfile.dat"/>
                <RestrictToRepacking name="rtr"/>
                <SeqprofConsensus name="seqprofcons"
min_aa_probability="1" probability_larger_than_current="0"
ignore_pose_profile_length_mismatch="1" keep_native="1" chain_num="0"
debug="1"/>

        </TASKOPERATIONS>

        <MOVERS>
                <RotamerTrialsMinMover name="sc_min"
scorefxn="talaris_full" task_operations="RIAAP_design,seqprofcons"/>
                <PackRotamersMover name="restore_cat_res"
scorefxn="talaris_full" task_operations="seqprofcons,No_RIAAP_design"/
>
                <AtomCoordinateCstMover name="chelating_res"
refrence_pdb="4zstA.pdb.gz" func_groups="1"
task_operations="RIAAP_design" coord_dev="0.01"/>
                <Subroutine name="splice_constraints"
xml_fname="splice_constraints_xsd.xml"/>
                <AddConstraintsToCurrentConformationMover
name="add_CA_cst" />
                <PackRotamersMover name="soft_design"
scorefxn="soft_rep_full"
task_operations="init,read_resfile,seqprofcons,no_repack_cat_Res,no_re
pack_cat_glu"/>
                <PackRotamersMover name="hard_design"
scorefxn="talaris_full"
task_operations="init,read_resfile,seqprofcons,no_repack_cat_Res,no_re
pack_cat_glu"/>
                <RotamerTrialsMinMover name="RTmin"
scorefxn="talaris_full"
task_operations="rtr,no_repack_cat_Res,no_repack_cat_glu"/>
                <TaskAwareMinMover name="soft_min"
scorefxn="soft_rep_full" chi="1" bb="1" jump="0"
```

```
task_operations="no_repack_cat_Res,no_repack_cat_glu" />
		<TaskAwareMinMover name="hard_min"
scorefxn="talaris_full" chi="1" bb="1" jump="0"
task_operations="no_repack_cat_Res,no_repack_cat_glu"/>
		<SaveAndRetrieveSidechains name="sars"/>
		<ParsedProtocol name="design_block">
			<Add mover_name="soft_design"/>
			<Add mover_name="soft_min"/>
			<Add mover_name="soft_design"/>
			<Add mover_name="hard_min"/>
			<Add mover_name="hard_design"/>
			<Add mover_name="hard_min"/>
			<Add mover_name="hard_design"/>
			Add mover_name=sars/>
			<Add mover_name="RTmin"/>
			<Add mover_name="RTmin"/>
			<Add mover_name="hard_min"/>
		</ParsedProtocol>
		<LoopOver iterations="4" mover_name="design_block"
name="iter4"/>
	</MOVERS>

	<FILTERS>
		<ScoreType confidence="0" name="stability_score_full"
score_type="total_score" scorefxn="talaris_full" threshold="0"/>
		<ScoreType confidence="0"
name="stability_without_pssm" score_type="total_score"
scorefxn="tal_no_pssm" threshold="0"/>
		<ScoreType confidence="0" name="stability_pure"
score_type="total_score" scorefxn="talaris_pure" threshold="0"/>
		<PackStat name="packstat" confidence="0"/>
		<Time name="timer"/>
		<DesignableResidues name="designable"
task_operations="No_RIAAP_design" designable="1" packable="0"/>
	</FILTERS>


	<PROTOCOLS>
		<Add mover_name="FSP"/>
		<Add mover_name="restore_cat_res"/>
		Add filter_name="designable"/>
		<Add mover_name="add_CA_cst"/>
		<Add mover_name="iter4"/>
		<Add filter_name="stability_score_full"/>
		<Add filter_name="stability_without_pssm"/>
		<Add filter_name="stability_pure"/>
		<Add filter_name="packstat"/>
	</PROTOCOLS>

</ROSETTASCRIPTS>
```

filterscan_auto_xsd.xml

```
<ROSETTASCRIPTS>

        <SCOREFXNS>

                <ScoreFunction name="talaris_full" weights="ref2015">

                        <Reweight scoretype="coordinate_constraint"
weight="0.4"/>
                        <Reweight scoretype="res_type_constraint"
weight="0.8"/>
                </ScoreFunction>

      </SCOREFXNS>
        <RESIDUE_SELECTORS>
<Chain name="chA" chains="A"/>
<Chain name="lig" chains="B"/>
<InterfaceByVector name="interF" grp1_selector="chA"
grp2_selector="lig"  cb_dist_cut="15" nearby_atom_cut="15"
vector_angle_cut="75" vector_dist_cut="9"/>
</RESIDUE_SELECTORS>


        <TASKOPERATIONS>


                <InitializeFromCommandline name="init"/>
                <SeqprofConsensus name="pssm_cutoff"
min_aa_probability="0" probability_larger_than_current="0"
convert_scores_to_probabilities="0" keep_native="1" debug="1"/>
                <RestrictIdentitiesAtAlignedPositions
name="no_repack_cat_Res" source_pdb="4zstA.pdb.gz"
prevent_repacking="1" resnums="267,196,167,23,21" keep_aas="HDKE"/
>keep cat arg
                <RestrictIdentitiesAtAlignedPositions
name="RIAAP_design" source_pdb="4zstA.pdb.gz"
resnums="135,267,196,167,23,21" keep_aas="HDKE"
design_only_target_residues="1"/>keep cat arg
<RestrictIdentitiesAtAlignedPositions name="no_repack_cat_glu"
source_pdb="4qsfA_ppk.pdb.gz" prevent_repacking="1" resnums="176"
keep_aas="HDKE"/>keep cat arg
                <DesignAround name="test" design_shell="1" resnums="2"
repack_shell="8" allow_design="1" resnums_allow_design="1"/>
        </TASKOPERATIONS>

        <MOVERS>
```

```
		<PackRotamersMover name="restore_cat_res" 
scorefxn="talaris_full" task_operations="RIAAP_design"/>
		<AddConstraintsToCurrentConformationMover 
name="add_CA_cst" />
		FavorSequenceProfile name="FSP" scaling="none" 
weight="1" pssm="%%pssm%%" scorefxns="talaris_full"/>
		<MinMover name="min_all" scorefxn="talaris_full" 
chi="1" bb="1" jump="0"/>
		Subroutine name="design" 
xml_fname="design_auto_xsd.xml"/>
		<RotamerTrialsMinMover name="sc_min" 
scorefxn="talaris_full" task_operations="RIAAP_design"/>
		<AtomCoordinateCstMover name="chelating_res" 
refrence_pdb="4zstA.pdb.gz" func_groups="1" 
task_operations="RIAAP_design" coord_dev="0.01"/>
		<Subroutine name="splice_constraints" 
xml_fname="splice_constraints_xsd.xml"/>
	</MOVERS>

	<FILTERS>
		<DesignableResidues name="designable" 
task_operations="init,pssm_cutoff,no_repack_cat_Res,no_repack_cat_glu" 
designable="1" packable="1"/>

		<ScoreType name="stability_score_full" 
scorefxn="talaris_full" score_type="total_score" threshold="0.0"/>
		<Delta name="delta_score_full" 
filter="stability_score_full" upper="1" lower="0" range="0.5"/>

		<Time name="timer"/>
		<FilterScan name="filter_scan" scorefxn="talaris_full" 
relax_mover="min_all" keep_native="1" 
task_operations="init,pssm_cutoff,no_repack_cat_Res,no_repack_cat_glu" 
delta_filters="delta_score_full" delta="true" resfile_name="resfile/
resfile.dat" report_all="1" delta_filter_thresholds="-0.75" 
score_log_file="%%pdb%%_score_full.log" dump_pdb="0"/>
	</FILTERS>

	<PROTOCOLS>
		<Add mover_name="restore_cat_res"/>
		<Add mover_name="add_CA_cst"/>
		<Add mover="splice_constraints"/>
		<Add filter="filter_scan"/>
		Add mover="design"/>
	</PROTOCOLS>

</ROSETTASCRIPTS>
```

splice_constraints_xsd.xml

```
<ROSETTASCRIPTS>a script for prepacking, redocking and minimizing a
pdb structure. The idea is to
relieve minor clashes that are often observed in PDBs.
Recommended command-line:
rostta_scripts -s  PDB  -parser:protocol refine_two_chain_pdb.xml -ex1
-ex2 -use_input_sc -database ~/minirosetta_database -
docking:fake_native
 <TASKOPERATIONS>
                <RestrictToRepacking name="rtr"/>
                SampleRotamersFromPDB name=template_chelating_res
add_rotamer=1 aligned_positions=22,24,137,170,198,255 debug=0/> //
These definitions are used for the E chelating residue of 1bf6
        </TASKOPERATIONS>

        <SCOREFXNS>
         <ScoreFunction name="talaris14" weights="ref2015">
                        <Reweight scoretype="res_type_constraint"
weight="0.5"/>
                </ScoreFunction>
        </SCOREFXNS>
        <FILTERS>
                <Ddg name="ddg" confidence="0"/>
                <Sasa name="sasa" confidence="0"/>
                <Rmsd name="rms" confidence="0"/>
        </FILTERS>
        <MOVERS>
<Splice name="splice_seqconstraints" scorefxn="talaris14"
add_sequence_constraints_only="1">tolrance=10>
      <Segments current_segment="blade4">
                                &lt;Segment name="frm1"
pdb_profile_match="pdb_profile_match" profiles="2vc5B:frm1_2vc5B.PSSM,
2vc5B:frm1_2vc5B.PSSM"/&gt;
                                &lt;Segment name="blade4"
pdb_profile_match="pdb_profile_match" profiles="%%blade4%%"/&gt;
                                &lt;Segment name="blade5"
pdb_profile_match="pdb_profile_match" profiles="%%blade5%%"/&gt;
                                &lt;Segment name="blade6"
pdb_profile_match="pdb_profile_match" profiles="%%blade6%%"/&gt;
                                &lt;Segment name="blade7"
pdb_profile_match="pdb_profile_match" profiles="%%blade7%%"/&gt;
                                &lt;Segment name="frm2"
pdb_profile_match="pdb_profile_match" profiles="2vc5B:frm2_2vc5B.PSSM,
2vc5B:frm2_2vc5B.PSSM"/&gt;
                        </Segments>
                </Splice>
```

```
        </MOVERS>
        <APPLY_TO_POSE>
        </APPLY_TO_POSE>
        <PROTOCOLS>
                <Add mover_name="splice_seqconstraints"/>
        </PROTOCOLS>
</ROSETTASCRIPTS>
```

splice_in_xsd.xml

```
<ROSETTASCRIPTS>
         <TASKOPERATIONS>
                <RestrictToRepacking name="rtr"/>

                <InitializeFromCommandline name="init"/>
                <SeqprofConsensus name="seqprofcons"
min_aa_probability="1" probability_larger_than_current="0"
ignore_pose_profile_length_mismatch="1" keep_native="1" debug="0"/>


                <RestrictIdentitiesAtAlignedPositions
name="RIAAP_no_des" source_pdb="4zstA.pdb"
design_only_target_residues="1" resnums="267,196,167,23,21"
keep_aas="HDGARNY"/>keep cat arg
<RestrictIdentitiesAtAlignedPositions name="RIAAP"
source_pdb="4zstA.pdb" design_only_target_residues="0"
resnums="267,196,167,23,21" keep_aas="HD"/>

        </TASKOPERATIONS>
        <SCOREFXNS>

                <ScoreFunction name="soft_rep_res_type_cst"
weights="soft_rep">
                        <Reweight scoretype="res_type_constraint"
weight="0.2"/>
                </ScoreFunction>

                <ScoreFunction name="TalarisCal_coordcst"
weights="ref2015">
                        <Reweight scoretype="res_type_constraint"
weight="0.2"/>
```

```
                        <Reweight scoretype="coordinate_constraint"
weight="1"/>
                        <Reweight scoretype="chainbreak" weight="10"/>
                </ScoreFunction>

                <ScoreFunction name="soft_rep_coordcst"
weights="soft_rep">
                        <Reweight scoretype="coordinate_constraint"
weight="0.06"/>
                </ScoreFunction>

                <ScoreFunction name="TalarisReg" weights="ref2015"/>
                <ScoreFunction name="talaris14" weights="ref2015">
                        <Reweight scoretype="res_type_constraint"
weight="0.5"/>
                        <Reweight scoretype="dihedral_constraint"
weight="10"/>
                        <Reweight scoretype="coordinate_constraint"
weight="10"/>
                        <Reweight scoretype="chainbreak" weight="10"/>
                </ScoreFunction>

                <ScoreFunction name="talaris2014_chainbreak"
weights="ref2015">
                        <Reweight scoretype="chainbreak" weight="1"/>
                </ScoreFunction>
        </SCOREFXNS>

        <FILTERS>
                <ScoreType name="chainbreak_val"
scorefxn="talaris2014_chainbreak" score_type="chainbreak"
threshold="0.2" confidence="1"/>
                <PackStat name="packstat" threshold="0.63" repeats="3"
confidence="0"/>
                <ScoreType name="total_score" score_type="total_score"
scorefxn="TalarisReg" threshold="1000000"/>
        </FILTERS>

        <MOVERS>
                <RotamerTrialsMinMover name="rtmin"
task_operations="rtr,init" scorefxn="TalarisCal_coordcst"/>

        <Splice name="splice_blade4"
torsion_database="2vc5B_blade4.db" use_sequence_profiles="1"
scorefxn="talaris14" ccd="0" thread_ala="0" repack_shell="100"
design_shell="100" template_file="%%start_pdb%%"
design_task_operations="init,seqprofcons" design="1" CG_const="0"
ignore_chain_break="1" debug="0" rtmin="1" min_seg="1"
delta_lengths="-1,0,1" equal_length="1" dbase_iterate="1">
                <Segments current_segment="blade4">
```

<Segment name="frm1"
pdb_profile_match="pdb_profile_match" profiles="2vc5B:frm1_2vc5B.PSSM,
2vc5B:frm1_2vc5B.PSSM"/>
<Segment name="blade4"
pdb_profile_match="pdb_profile_match" profiles="%%blade4%%"/>
<Segment name="blade5"
pdb_profile_match="pdb_profile_match" profiles="%%blade5%%"/>
<Segment name="blade6"
pdb_profile_match="pdb_profile_match" profiles="%%blade6%%"/>
<Segment name="blade7"
pdb_profile_match="pdb_profile_match" profiles="%%blade7%%"/>
<Segment name="frm2"
pdb_profile_match="pdb_profile_match" profiles="2vc5B:frm2_2vc5B.PSSM,
2vc5B:frm2_2vc5B.PSSM"/>
</Segments>
</Splice>

<Splice name="splice_blade5"
torsion_database="2vc5B_blade5.db" use_sequence_profiles="1"
scorefxn="talaris14" ccd="0" thread_ala="0" repack_shell="100"
design_shell="100" template_file="%%start_pdb%%"
design_task_operations="init,seqprofcons" design="1" CG_const="0"
ignore_chain_break="1" debug="0" rtmin="1" min_seg="1"
delta_lengths="-1,0,1" equal_length="1" dbase_iterate="1">
<Segments current_segment="blade5">
<Segment name="frm1"
pdb_profile_match="pdb_profile_match" profiles="2vc5B:frm1_2vc5B.PSSM,
2vc5B:frm1_2vc5B.PSSM"/>
<Segment name="blade4"
pdb_profile_match="pdb_profile_match" profiles="%%blade4%%"/>
<Segment name="blade5"
pdb_profile_match="pdb_profile_match" profiles="%%blade5%%"/>
<Segment name="blade6"
pdb_profile_match="pdb_profile_match" profiles="%%blade6%%"/>
<Segment name="blade7"
pdb_profile_match="pdb_profile_match" profiles="%%blade7%%"/>
<Segment name="frm2"
pdb_profile_match="pdb_profile_match" profiles="2vc5B:frm2_2vc5B.PSSM,
2vc5B:frm2_2vc5B.PSSM"/>
</Segments>
</Splice>

<Splice name="splice_blade6" torsion_database="2vc5B_blade6.db"
use_sequence_profiles="1" scorefxn="talaris14" ccd="0" thread_ala="0"
repack_shell="100" design_shell="100" template_file="%%start_pdb%%"
design_task_operations="init,seqprofcons" design="1" CG_const="0"
ignore_chain_break="1" debug="0" rtmin="1" min_seg="1"
delta_lengths="-1,0,1" equal_length="1" dbase_iterate="1">
<Segments current_segment="blade6">
<Segment name="frm1"

```
pdb_profile_match="pdb_profile_match" profiles="2vc5B:frm1_2vc5B.PSSM,
2vc5B:frm1_2vc5B.PSSM"/>
                                &lt;Segment name="blade4"
pdb_profile_match="pdb_profile_match" profiles="%%blade4%%"/>
                                &lt;Segment name="blade5"
pdb_profile_match="pdb_profile_match" profiles="%%blade5%%"/>
                                &lt;Segment name="blade6"
pdb_profile_match="pdb_profile_match" profiles="%%blade6%%"/>
                                &lt;Segment name="blade7"
pdb_profile_match="pdb_profile_match" profiles="%%blade7%%"/>
                                &lt;Segment name="frm2"
pdb_profile_match="pdb_profile_match" profiles="2vc5B:frm2_2vc5B.PSSM,
2vc5B:frm2_2vc5B.PSSM"/>
                        </Segments>
                </Splice>

<Splice name="splice_blade7" torsion_database="2vc5B_blade7.db"
use_sequence_profiles="1" scorefxn="talaris14" ccd="0" thread_ala="0"
repack_shell="100" design_shell="100" template_file="%%start_pdb%%"
design_task_operations="init,seqprofcons" design="1" CG_const="0"
ignore_chain_break="1" debug="0" rtmin="1" min_seg="1"
delta_lengths="-1,0,1" equal_length="1" dbase_iterate="1">
                <Segments current_segment="blade6">
                                &lt;Segment name="frm1"
pdb_profile_match="pdb_profile_match" profiles="2vc5B:frm1_2vc5B.PSSM,
2vc5B:frm1_2vc5B.PSSM"/>
                                &lt;Segment name="blade4"
pdb_profile_match="pdb_profile_match" profiles="%%blade4%%"/>
                                &lt;Segment name="blade5"
pdb_profile_match="pdb_profile_match" profiles="%%blade5%%"/>
                                &lt;Segment name="blade6"
pdb_profile_match="pdb_profile_match" profiles="%%blade6%%"/>
                                &lt;Segment name="blade7"
pdb_profile_match="pdb_profile_match" profiles="%%blade7%%"/>
                                &lt;Segment name="frm2"
pdb_profile_match="pdb_profile_match" profiles="2vc5B:frm2_2vc5B.PSSM,
2vc5B:frm2_2vc5B.PSSM"/>
                        </Segments>
                </Splice>

                <ClearConstraintsMover name="clear_constraints"/>
                <Subroutine name="splice_constraints"
xml_fname="splice_constraints_xsd.xml"/>
                Subroutine name="splice_blade4"
xml_fname="splice_in_blade4_db_itterate.xml"/>
                Subroutine name="splice_blade5"
xml_fname="splice_in_blade5_db_itterate.xml"/>
                Subroutine name="splice_blade6"
xml_fname="splice_in_blade6_db_itterate.xml"/>
```

```
         <AtomCoordinateCstMover name="chelating_res"
refrence_pdb="4zstA.pdb" func_groups="1"
task_operations="RIAAP_no_des" coord_dev="0.01"/>
        <PackRotamersMover name="ppk" scorefxn="talaris14"
task_operations="seqprofcons" />
                <ParsedProtocol name="splice_menu"
mode="single_random"> Sampling blade_7-8 more becasue it's more
variable
                        <Add mover="splice_blade4"/>
                        <Add mover="splice_blade5"/>
                        <Add mover="splice_blade6"/>
                        <Add mover="splice_blade7"/>
                </ParsedProtocol>



                <GenericSimulatedAnnealer name="annealer"
mover_name="splice_menu" filter_name="total_score" trials="30"
preapply="0" />
                <PackRotamersMover name="design_talarisCal"
scorefxn="TalarisCal_coordcst" task_operations="init,seqprofcons"/>
                <PackRotamersMover name="design_softrep"
scorefxn="soft_rep_coordcst" task_operations="init,seqprofcons"/>

                <TaskAwareMinMover name="soft_min"
scorefxn="soft_rep_coordcst" bb="0" jump="0" chi="1"
task_operations="rtr,init"/> do not minimize bb
                <TaskAwareMinMover name="soft_min_all"
scorefxn="soft_rep_coordcst" bb="1" jump="0" chi="1"/> minimize all
but don't minimize chain2 backbone because it could contain tails and
holes
                <TaskAwareMinMover name="hard_min"
scorefxn="TalarisCal_coordcst" bb="0" jump="0" chi="1"
task_operations="rtr,init"/>



                <ParsedProtocol name="post_splice_refine">
                        <Add mover="clear_constraints"/>        //
remove any residual constraints from splice mover
                        <Add mover="splice_constraints"/>       //re-
institute the sequence constraints
                        <Add mover="chelating_res"/>
                        <Add mover="soft_min"/>
                        <Add mover="hard_min"/>
                        <Add mover="design_softrep"/>
                        <Add mover="soft_min"/>
                        <Add mover="hard_min"/>
                        <Add mover="design_talarisCal"/>
```

```
				<Add mover="rtmin"/>
				<Add mover="hard_min"/>
			</ParsedProtocol>
		</MOVERS>

		<PROTOCOLS>
			<Add mover="chelating_res"/>
			<Add mover="annealer"/>
			<Add mover="post_splice_refine"/>
			<Add filter="packstat"/>

		</PROTOCOLS>
	</ROSETTASCRIPTS>
```
